# Peptide-Mediated Synthesis of Zeolitic Imidazolate Framework-8: Effect of Molecular Hydrophobicity, Charge Number and Charge Location

**DOI:** 10.3390/nano11102665

**Published:** 2021-10-12

**Authors:** Maozhang Tian, Xi Chen, Qun Zhang, Xinyuan Zou, Desheng Ma, Jiaming Xuan, Wentao Wang, Meiwen Cao

**Affiliations:** 1 State Key Laboratory of Enhanced Oil Recovery, Research Institute of Petroleum Exploration and Development, CNPC, Beijing 100083, China; mztian@petrochina.com.cn (M.T.); xchen63@petrochina.com.cn (X.C.); zhangqun1980@petrochina.com.cn (Q.Z.); zouxy2016@petrochina.com.cn (X.Z.); mads6@petrochina.com.cn (D.M.); 2 State Key Laboratory of Heavy Oil Processing, Department of Biological and Energy Chemical Engineering, College of Chemical Engineering, China University of Petroleum (East China), 66 Changjiang West Road, Qingdao 266580, China; mr_xuanjam@163.com; 3 Department of Radiochemistry, China Institute of Atomic Energy, Beijing 102413, China; wentaowang001@163.com

**Keywords:** amphiphilic peptide, zeolitic imidazolate framework, crystal growth

## Abstract

Three amphiphilic peptides with varied molecular hydrophobicity, charge number and charge location have been designed as regulators to modulate the crystal growth of zeolitic imidazolate framework-8 (ZIF-8). All three peptides can interact with ZIF-8 to inhibit {100} facet growth and produce truncated cubic crystals. The peptide’s molecular hydrophobicity plays a dominant role in defining the final morphology and size of the ZIF-8 crystals. The peptides with less charge and higher hydrophobicity can promote nuclei formation and crystal growth to give smaller ZIF-8 crystals. However, the charge located in the center of the molecular hydrophobic region has little effect on the crystal nucleation and growth due to the shielding of its charge by molecular aggregation. The study provides insights into the effect of molecular charge and hydrophobicity on ZIF-8 crystal growth and is helpful for guiding the molecular design for regulating the synthesis of metal-organic framework materials.

## 1. Introduction

Metal-organic frameworks (MOFs) are a novel class of crystallized porous materials that are self-assembled by metal clusters as connecting points and organic ligands as bridging molecules to form a two- or three-dimensional framework through coordination bonds [1,2,3,4,5,6,7]. MOFs have attracted great interests in recent years by having remarkable features such as adjustable structure, high porosity, large specific surface area, good modifiability and excellent stability and have been used widely in many areas, for example, adsorption [8,9,10,11,12,13], catalysis [14,15,16,17,18], sensing [19,20,21,22,23], drug delivery [24,25,26,27,28,29,30] and gas storage and separation [31,32,33], etc. Besides the intrinsic properties of MOFs, their morphology and size also play crucial roles for their practical applications. Therefore, researchers have put a lot of effort into developing strategies for controlling the morphology and size of MOFs [34,35,36,37,38,39,40,41,42,43].

Zeolitic imidazolate framework-8 (ZIF-8) is one typical kind of MOF that is comprised of zinc ions and 2-methylimidazole [34,44,45,46]. It is characterized by high porosity and a large surface area and has great advantages in the encapsulation and delivery of functional species [13,16,47,48,49,50]. Moreover, it has high biocompatibility, excellent stability under physiological conditions and good responsiveness towards acidic conditions. These merits make ZIF-8 an ideal carrier for drug delivery and release in disease treatment [50,51,52,53]. To optimize the performance of ZIF-8 nanoparticles in practical applications, various methods have been applied to modulate their shape and size [35,38]. One common strategy is to modulate the shape and structure of ZIF-8 by adjusting the solvent composition for ZIF-8 synthesis. For example, Yang et al. and Hadi et al. produced ZIF-8 nanoparticles with varied dimensions, structures and sizes by adjusting the methanol/water ratio of the reaction solvent [54,55]. Another effective strategy for controlling the shape and size of ZIF-8 is to regulate its crystallization and growth by the addition of surfactants in the reaction solution. Pan et al. successfully synthesized ZIF-8 crystals with a distinct structure and size by using various surfactants (e.g., CTAB, CTAC, STAC and TPABr) as capping agents to modulate the facet growth of ZIF-8 [35]. Zhao et al. synthesized ZIF-8 nanocrystals with varied morphology and size by using the Brij reverse micelles as spatially constrained nanoreactors [56]. However, though these methods can achieve successful control over the shape and size of ZIF-8, the obtained ZIF-8 nanomaterials may not be suitable for biomedical applications because some toxic species are introduced into the materials during synthesis.

Recently, we report a green method for the synthesis of biocompatible ZIF-8 nanoparticles by using amphiphilic peptides as capping agents [57]. The use of peptides as capping agents can not only realize efficient control over the shape and size of ZIF-8 crystals but can also introduce stimuli-sensitive groups to the ZIF-8 surface to enable specific biofunctionalilties. Moreover, by having high biocompatibility and low toxicity [57,58,59,60], peptide-incorporated ZIF-8 crystals will be more suitable for biomedical applications. In the above work, we mainly demonstrated the effect of molecular hydrophobicity on the regulation of the ZIF-8 crystal growth. Here, we further present the effects of molecular hydrophobicity, charge number and charge location of the peptide molecule on ZIF-8 crystal growth, aiming to provide guiding principles for molecular design to modulate the morphology and size of MOFs.

## 2. Materials and Methods

### 2.1. Materials

All peptides with >96% purity were synthesized by Shanghai Top-Peptide Biotechnology Co. Ltd. 2-methylimidazole was from J&K Scientific Ltd. (Beijing, China). Zn(CH_3_COO)_2_∙2H_2_O was obtained from Sinopharm Chemical Reagent Co., Ltd. (Beijing, China). The water with a resistivity of 18.2 MΩ·cm was treated by an ultrapure water processing system (Milli-Q Biocel) and was used in all of the experiments.

### 2.2. ZIF-8 Synthesis

In a typical synthesis process, 0.1068 g Zn(CH_3_COO)_2_∙2H_2_O was dissolved in 40 mL water, and then 4.0 g 2-methylimidazole was added to the solution under constant stirring by RHDS25 equipment (IKA^®^, Kunshan, China) at room temperature. The mixed solution was incubated at room temperature for about 24 h. The mixed solution turned milky during incubation, indicating the formation of ZIF-8 crystals. After incubation, the ZIF-8 crystals were collected by centrifugation at 10,000 rpm for 10 min and the sediments were washed with pure water three times, which were then freeze-dried to obtain the final products.

### 2.3. Peptide-Mediated ZIF-8 Synthesis

First, the stock solutions of the two reactive species were prepared separately by dissolving either 0.2136 g of Zn(CH_3_COO)_2_ or 8.0 g of 2-methylimidazole in 40 mL of Milli-Q water. Then, a defined amount of peptide powder (76.4 mg for ***RK***, 80.7 mg for ***K-RK*** and 93.4 mg for ***RKRK***) was added to the Zn(CH_3_COO)_2_ solution of 30 mL while stirring to prepare the peptide solution of 2.0 mM. Next, 30 mL of the above 2-methylimidazole solution was added to the as-prepared Zn(CH_3_COO)_2_/peptide mixed solution under constant stirring (RHDS25, IKA^®^) at 25 °C. After incubation for about 24 h, the mixed solution turned milky. The ZIF-8 crystals were then collected by centrifugation at 10,000 rpm for 10 min and the sediments were washed with water three times. The products were then freeze-dried to obtain the ZIF-8 powder.

### 2.4. Characterization

Transmission electron microscopy (TEM) images of the ZIF-8 nanoparticles were taken with JEOL JEM-1400 equipment (Tokyo, Japan) operating at 120 kV. Scanning electron microscopy (SEM) measurements were performed on a FEI QUANTA FEG250 instrument (Hillsboro, OR, USA) operated at an accelerating voltage of 5.0 kV. Powder X-ray diffraction (XRD) data in the range of 2θ = 5–76° were collected on an X’Pert PRO MPD diffractometer (Almelo, Holland) with Cu Kα (λ = 0.154 nm) source at a scan rate of 5°/min. Fourier-transform infrared spectroscopy (FT-IR) samples were prepared by KBr pellet method and the transmission spectra were obtained at room temperature with a Nicolet iS5 (Thermo Fisher Scientific, Bremen, Germany) instrument in the range of 4000–400 cm^−1^. Nitrogen physisorption isotherms were measured with an automatic Tristar II 3020 volumetric adsorption apparatus (Micromeritics Instruments, Norcross, GA, USA). Dynamic light scattering (DLS) and zeta potential (ζ) measurements were performed with a Nano-ZS instrument (ZEN3600, Malvern Instruments, Worcestershire, UK). The solution absorption at 400 nm was obtained with a UV-2450 spectrophotometer (Pharma Spec, Shimadzu, Kyoto, Japan) for turbidity evaluation. X-ray photoelectron spectroscopy (XPS) analysis was performed with an Escalab 250Xi electron spectrometer (Thermo Scientific, Waltham, MA, USA) with a mono Al Ka X-ray source.

## 3. Results

### 3.1. Peptide Molecular Design

**Figure 1:**
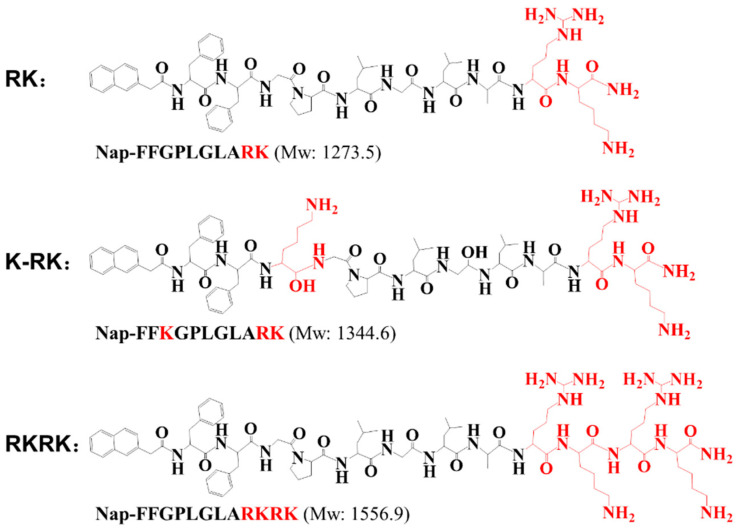
Molecular structures of the peptides used as capping agents for ZIF-8 synthesis.

Figure 1 shows the molecular structures and molecular weights of the three peptides that are used as capping agents for ZIF-8 synthesis. The peptides have a purity of >96% and the impurities may come from peptide fragments that have not reacted completely and hence trifluoroacetate left during peptide synthesis. They are all amphiphilic molecules with the same hydrophobic residues but different hydrophilic charged residues. According to the number and location of the charged residues in the molecules, the three peptides are termed ***RK***, ***K-RK*** and ***RKRK***, respectively. ***RK*** and ***RKRK*** are surfactant-like with two segments. Their hydrophobic segment is the same as Nap-FFGPLGLA- [61,62,63], while they have a different number of positive residues (-RK- or -RKRK-) to form the hydrophilic segments. In contrast, by having one lysine residue located in the middle of the hydrophobic region, ***K-RK*** shows four distinct segments in its molecular structure. Such a molecular design produces three peptide molecules with varied molecular hydrophobicity, charge and charge distribution, which are used as regulators for ZIF-8 synthesis.

### 3.2. Peptide-Mediated ZIF-8 Synthesis

**Figure 2:**
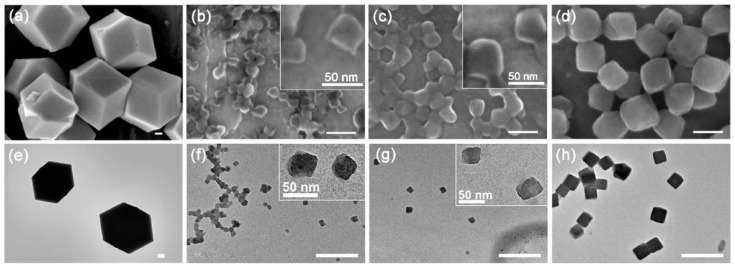
SEM (**a**–**d**) and TEM (**e**–**h**) images of the ZIF-8 nanoparticles synthesized in the absence (**a**,**e**) or presence of ***RK*** (**b**,**f**), ***K-RK*** (**c**,**g**) and ***RKRK*** (**d**,**h**), respectively. The scale bars for the SEM images are 100 nm. The scale bars for the TEM images are 200 nm. The insets of images (**b**,**c**,**f**,**g**) are the corresponding high-resolution images. The peptide concentration is 1.0 mM in all cases.

**Figure 3:**
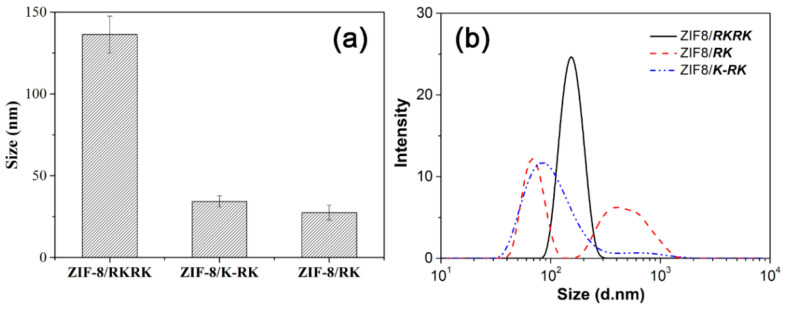
Size distribution of the ZIF-8 crystals prepared in the presence of ***RKRK***, ***K-RK*** and ***RK***, (**a**) TEM-derived sizes, (**b**) DLS size distribution.

Figure 2 presents the SEM and TEM images of the ZIF-8 crystals prepared in the absence or presence of different peptides at 1.0 mM. The ZIF-8 crystals prepared in water with the absence of any peptide displayed a typical rhombic dodecahedra shape (Figure 2a,e) with the average size > 1.0 μm, being consistent with the results reported previously [35,57]. In contrast, the morphology and size of the ZIF-8 crystal were greatly modified with the addition of peptides in the synthesis solution as regulators. The three peptides all produced ZIF-8 crystals with basic truncated cubic profiles. However, the ZIF-8 produced by ***RKRK*** were the most cubic, while the ZIF-8 produced by ***RK*** and ***K-RK*** showed a great level of shape deformation, as can be clearly observed from the inset high-resolution images of Figure 2b,c,f,g. Their mean sizes (obtained from the TEM images) were ca. 137 nm, 28 nm and 32 nm in the cases of ***RKRK***, ***RK*** and ***K-RK***, respectively (Figure 3a). DLS measurements were also performed to characterize the size of the ZIF-8 crystals obtained in the presence of different peptides. In the case of ***RKRK***, the DLS profile showed a single peak with a size distribution similar to that derived from TEM (Figure 3b). However, in the cases of ***RK*** and ***K-RK***, the DLS results gave two size distribution bands, one at the smaller size region and the other at the larger size region. The smaller size distribution was ascribed to the dispersed ZIF-8 particles but was larger than those obtained from TEM. The reason may be that the ZIF-8 particles had a hydration layer in water, which increased the hydrodiameters measured by DLS, whereas, the larger size distribution can be interpreted by the aggregation of the ZIF-8 particles, as can be confirmed by the SEM and TEM images of Figure 2b,c,f. The above results show clearly that the morphology and size of the ZIF-8 crystals were significantly modified by the addition of peptides in the synthesis solutions.

**Figure 4:**
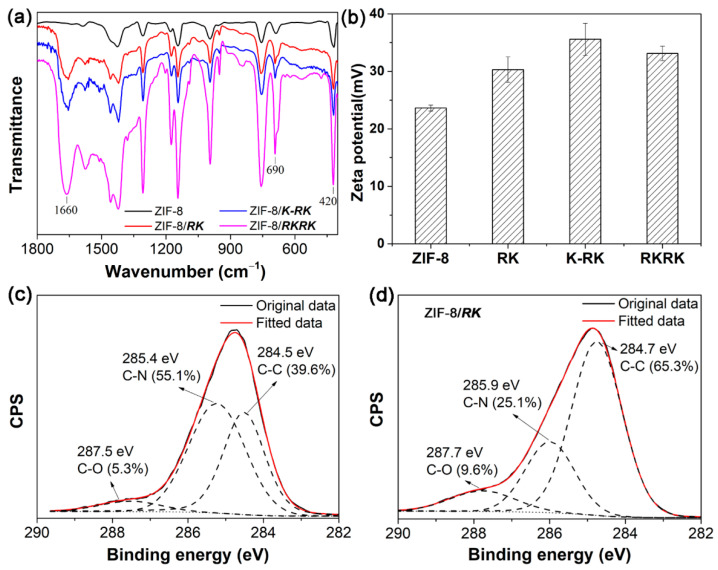
FTIR spectra (**a**) and zeta potential values (**b**) of the ZIF-8 crystals prepared in pure water or in the presence of ***RKRK***, ***K-RK*** and ***RK***, respectively. C 1s XPS spectra of the ZIF-8 prepared in pure water (**c**) and in the presence of peptide ***RK*** (**d**).

Figure 4a shows the FTIR spectra of the ZIF-8 crystals prepared in the presence of ***RKRK***, ***K-RK*** and ***RK***, respectively. The spectrum of ZIF-8 prepared in pure water is also given for comparison. Compared to the sample prepared in water, the spectra of the ZIF-8 prepared with peptides show an additional strong peak at ~1660 cm^−1^, which is the peptidic amide I band [57]. The results indicate that the peptide molecules were incorporated into the ZIF-8 crystals. Moreover, two bands at 690 and 420 cm^−1^ were observed for all samples, which may be ascribed to stretching vibrations of Zn-O and Zn-N bonds, respectively. Such results indicate that the peptide molecules were adsorbed on the surface of ZIF-8 crystals [57]. Moreover, the zeta potential results (Figure 4b) show that, compared to ZIF-8 prepared in pure water, the samples prepared in the presence of peptides gave larger positive values, also indicating the adsorption of positively charged peptide molecules on the surface of ZIF-8 crystals. Furthermore, the C 1s XPS spectra of ZIF-8 and ZIF-8/***RK*** are shown in Figure 4c,d. For ZIF-8 prepared in water, the fitted data of C 1s gave contents of C-C (284.5 eV) 39.6%, C-N (285.4 eV) 55.1% and C=O (287.5 eV) 5.3%, respectively. Whilst for ZIF-8 prepared in the presence of peptide ***RK***, the C-C and C=O components increased greatly to 65.3% and 9.6%, respectively, and the C-N component decreased to 25.1%. The results also confirm the adsorption of peptide molecules on ZIF-8, which changes the carbon composition of the ZIF-8/peptide complexes [57].

**Figure 5:**
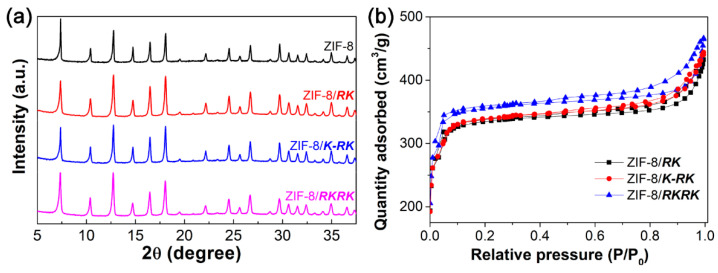
XRD patterns (**a**) and N_2_ adsorption isotherms (**b**) of the ZIF-8 crystals prepared in pure water or in the presence of each peptide.

Figure 5a shows the XRD patterns of the ZIF-8 crystals produced in different cases. As can be observed, the XRD spectra of ZIF-8 crystals prepared in the presence of peptides are nearly identical to that of ZIF-8 prepared in pure water. The fact that the peptides did not affect the crystallinity of ZIF-8 crystals indicates that the peptides probably bind on the ZIF-8 surface rather than incorporating them into the internal crystal framework. N_2_ adsorption isotherms were further used to obtain information on the surface area and mesoporous volume of the ZIF-8 crystals (Figure 5b). Similar to our recent findings, the ZIF-8 crystals prepared in the presence of each peptide all show a type I sorption profile [57], that is, the crystals have microporous structures. The specific surface area (S_BET_) and mesoporous volume (V_micro_) of the ZIF-8 crystals prepared in the presence of each peptide are 1407 m^2^·g^−1^ and 0.50 cm^−3^·g^−1^ for ***RK***, 1416 m^2^·g^−1^ and 0.52 cm^−3^·g^−1^ for ***K-RK*** and 1452 m^2^·g^−1^ and 0.57 cm^−3^·g^−1^ for ***RKRK***, respectively.

**Figure 6:**
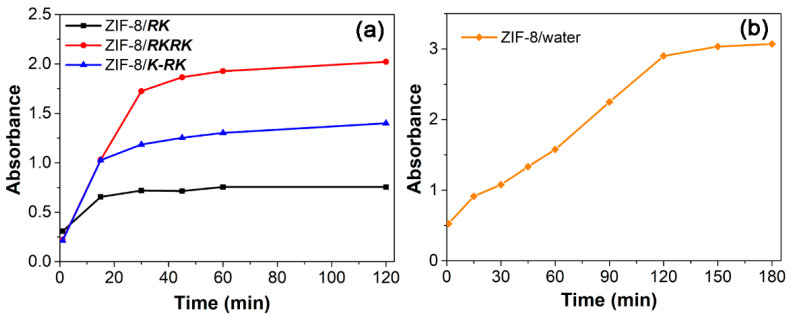
Variation of the solution turbidity with the elapse of reaction time during synthesis of ZIF-8 in the presence (**a**) or absence (**b**) of different peptides.

**Figure 7:**
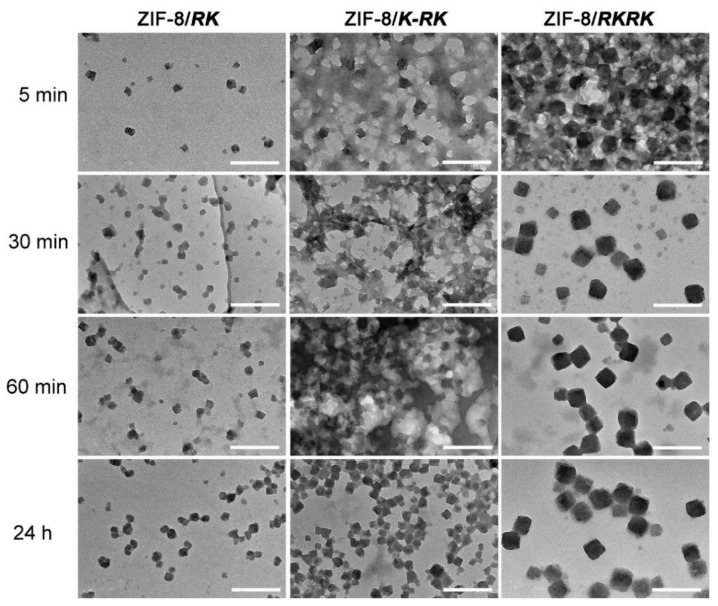
TEM characterization showing the morphology change of the ZIF-8 crystals prepared in the presence of each peptide at varied reaction times. Scale bar represents 200 nm for all images.

The dynamic process of ZIF-8 growth in the presence or absence of different peptides was then followed by both turbidity and TEM measurements. Figure 6 shows the evolution of the solution turbidity at 400 nm as a function of reaction time. All curves show an initial sharp increase in turbidity that indicates fast nuclei formation and crystal growth, which is followed by a plateau region that indicates the equilibrium of crystal growth. The time points for reaching crystal growth equilibrium are ~15, 22 and 40 min in the cases of ***RK***, ***K-RK*** and ***RKRK***, respectively, clearly showing that the nucleation and crystal growth speed can be ordered as ***RK*** > ***K-RK*** > ***RKRK***. However, the equilibrium turbidity value gave a reverse order, that is, ***RK*** < ***K-RK*** < ***RKRK***, indicating differences in particle size and/or particle number in each case. On the other hand, for the turbidity vs. time profile of ZIF-8 growth in pure water, the equilibrium time was about 120 min, which is much larger than those of peptide-mediated ZIF-8 growth. And, the equilibrium turbidity was around 3, which is also larger than those produced in the presence of peptides. The results demonstrate that the peptides can promote the nucleation and growth of ZIF-8 crystals while inhibiting their size growth. The reason may be that the peptide self-assembled structures probably acted as nucleation sites and capping agents to mediate ZIF-8 nuclei formation and growth, which resulted in many more ZIF-8 crystals with smaller sizes. Figure 7 shows the morphology evolution of the ZIF-8 crystals at varied reaction times in different cases. At 5 min, many tiny particles with irregular shapes can be observed in each case, which should be the nuclei formed in the early stage. Then, the particle size increased gradually with the elapse of time until the final equilibrium size was reached, clearly showing the crystal growth stage. Once again, we can see clearly the differences in morphology and size of the ZIF-8 crystals in each case, which were consistent with the results shown in Figure 2 and Figure 3, confirming the reproducibility of the peptide-regulated ZIF-8 synthesis. The larger particle size in the case of ***RKRK*** corresponds well to its largest turbidity value (Figure 6). However, the particles give a similar size in the cases of ***RK*** and ***K-RK*** while their turbidity value shows a significant difference. These results may be interpreted by the difference in particle number and/or particle aggregation propensity in the two cases. We should also note that in some images, especially those obtained at the early stage of nucleation and crystal growth, the ZIF-8 particles were usually attached to some network structures, which should be the peptide aggregates. This result indicates that the peptides can bind on the ZIF-8 surface to affect its nucleation and growth.

### 3.3. Discussion on the Effect of Molecular Hydrophobicity, Charge Number and Charge Location

The above results clearly demonstrate that the peptide molecules can work as regulators to modulate the morphology and size of the ZIF-8 crystals, which depends greatly on the peptide molecular structure [57]. In the present case, the peptide molecules were rationally designed to have different hydrophobicity, charge number and charge location. First, all three molecules can produce ZIF-8 crystals with truncated cubic profiles. This can be interpreted by Wulff’s rule, that is, the final crystal shape depends on the slow-growing face. Here, the peptides can bind on the ZIF-8 surface to inhibit the {100} facet growth so as to result in final cubic crystals with six {100} faces. Second, because the three molecules have the same hydrophobic residues but different charged residues, the overall molecular hydrophobicity is inversely correlated with the charge numbers and can be ordered as ***RK*** > ***K-RK*** > ***RKRK***. This order is the same with the order of the nucleation and growth speed of the ZIF-8 particles and the reverse order of the particle sizes in each case. These results indicate that molecular hydrophobicity plays crucial roles in regulating the nucleation and growth of ZIF-8 crystals. The peptide with higher hydrophobicity (e.g., ***RK***) will promote nuclei formation at the early stage. With more nuclei being formed, more species (2-methylimidazole and Zn^2+^) for ZIF-8 synthesis will be consumed and fewer are left for the following crystal growth stage. Therefore, a faster nucleation and crystal growth speed but a smaller crystal size will be the case [64,65,66]. This interprets well the effect of molecular hydrophobicity and charge number on ZIF-8 synthesis. Furthermore, for ***RK*** and ***K-RK***, their molecular hydrophobicity differs greatly whilst the final ZIF-8 crystals show little difference in size. The results indicate that a positive lysine residue in the middle of the hydrophobic region does not affect greatly the interactions of the peptide molecule with ZIF-8. The reason may be that the middle positive charge is buried inside during peptide aggregation and self-assembly and therefore its function is blocked. One may also conclude that the charge location rather than charge number plays more important roles in affecting the ZIF-8 crystal growth.

## 4. Conclusions

In conclusion, the effects of molecular hydrophobicity, charge number and charge location on the morphology and size of the ZIF-8 crystals have been investigated by rationally designing three amphiphilic peptides as regulators for ZIF-8 synthesis. The peptide molecular hydrophobicity plays a dominant role in defining the final morphology of the ZIF-8 crystals. All three peptides of ***RK***, ***K-RK*** and ***RKRK*** can bind on the ZIF-8 surface to inhibit the {100} facet growth and result in the production of truncated cubic crystals. The peptides with fewer charges and higher hydrophobicity promote nuclei formation and crystal growth to give smaller ZIF-8 crystals. The charge located in the hydrophobic region of the peptide molecule has little effect on the crystal nucleation and growth because its effect will be shielded due to molecular aggregation. The study provides insights into the effect of molecular charge and hydrophobicity on ZIF-8 crystal growth, which is helpful for guiding the molecular design for regulating ZIF-8 synthesis.

## Data Availability

The data presented in this study are available on request from the corresponding author. The data are not publicly available due to limited web resource.
